# Brain Perfusion Mediates the Relationship Between miRNA Levels and Postural Control

**DOI:** 10.1093/texcom/tgaa078

**Published:** 2020-10-26

**Authors:** Yufen Chen, Amy A Herrold, Zoran Martinovich, Sumra Bari, Nicole L Vike, Anne J Blood, Alexa E Walter, Jaroslaw Harezlak, Peter H Seidenberg, Manish Bhomia, Barbara Knollmann-Ritschel, Khrystyna Stetsiv, James L Reilly, Eric A Nauman, Thomas M Talavage, Linda Papa, Semyon Slobounov, Hans C Breiter, for the Concussion Neuroimaging Consortium

**Affiliations:** Center for Translational Imaging, Department of Radiology, Feinberg School of Medicine, Northwestern University, Chicago, IL 60611, USA; Edward Hines Jr., VA Hospital, Research Service, Hines, IL 60141, USA; Warren Wright Adolescent Center, Department of Psychiatry and Behavioral Sciences, Feinberg School of Medicine, Northwestern University, Chicago, IL 60611, USA; Mental Health Services and Policy Program, Department of Psychiatry and Behavioral Sciences, Northwestern University Feinberg School of Medicine, Chicago, IL 60611, USA; Warren Wright Adolescent Center, Department of Psychiatry and Behavioral Sciences, Feinberg School of Medicine, Northwestern University, Chicago, IL 60611, USA; School of Electrical and Computer Engineering, Purdue University, West Lafayette, IN 47907, USA; Warren Wright Adolescent Center, Department of Psychiatry and Behavioral Sciences, Feinberg School of Medicine, Northwestern University, Chicago, IL 60611, USA; School of Electrical and Computer Engineering, Purdue University, West Lafayette, IN 47907, USA; Weldon School of Biomedical Engineering, Purdue University, West Lafayette, IN 47907, USA; Mood and Motor Control Laboratory, Departments of Neurology and Psychiatry, Massachusetts General Hospital and Harvard Medical School, Boston, MA 02129, USA; Laboratory of Neuroimaging and Genetics, Department of Psychiatry, Massachusetts General Hospital and Harvard Medical School, Boston, MA 02129, USA; Martinos Center for Biomedical Imaging, Department of Radiology, Massachusetts General Hospital and Harvard Medical School, Boston, MA 02129, USA; Department of Kinesiology, Pennsylvania State University, University Park, PA 16802, USA; Department of Epidemiology and Biostatistics, Indiana University, Bloomington, IN 47405, USA; Departments of Orthopaedics & Rehabilitation and Family & Community Medicine, Penn State College of Medicine, Hershey, PA 17033, USA; Department of Pathology, Uniformed Services University of the Health Sciences, Bethesda, MD, USA; Department of Pathology, Uniformed Services University of the Health Sciences, Bethesda, MD, USA; Warren Wright Adolescent Center, Department of Psychiatry and Behavioral Sciences, Feinberg School of Medicine, Northwestern University, Chicago, IL 60611, USA; Warren Wright Adolescent Center, Department of Psychiatry and Behavioral Sciences, Feinberg School of Medicine, Northwestern University, Chicago, IL 60611, USA; Weldon School of Biomedical Engineering, Purdue University, West Lafayette, IN 47907, USA; Department of Basic Medical Sciences, Purdue University, West Lafayette, IN 47907, USA; School of Mechanical Engineering, Purdue University, West Lafayette, IN 47907, USA; School of Electrical and Computer Engineering, Purdue University, West Lafayette, IN 47907, USA; Weldon School of Biomedical Engineering, Purdue University, West Lafayette, IN 47907, USA; Department of Emergency Medicine, Orlando Regional Medical Center, Orlando, FL, USA; Department of Kinesiology, Pennsylvania State University, University Park, PA 16802, USA; Warren Wright Adolescent Center, Department of Psychiatry and Behavioral Sciences, Feinberg School of Medicine, Northwestern University, Chicago, IL 60611, USA; Laboratory of Neuroimaging and Genetics, Department of Psychiatry, Massachusetts General Hospital and Harvard Medical School, Boston, MA 02129, USA

**Keywords:** football athletes, head acceleration events, microRNAs, postural control, regional cerebral blood flow

## Abstract

Transcriptomics, regional cerebral blood flow (rCBF), and a virtual reality-based spatial motor task were integrated using mediation analysis in a novel demonstration of “imaging omics.” Data collected in National Collegiate Athletic Association (NCAA) Division I football athletes cleared for play before in-season training showed significant relationships in 1) elevated levels of miR-30d and miR-92a to elevated putamen rCBF, 2) elevated putamen rCBF to compromised Balance scores, and 3) compromised Balance scores to elevated microRNA (miRNA) levels. rCBF acted as a consistent mediator variable (Sobel’s test *P* < 0.05) between abnormal miRNA levels and compromised Balance scores. Given the involvement of these miRNAs in inflammation and immune function and that vascular perfusion is a component of the inflammatory response, these findings support a chronic inflammatory model in these athletes with 11 years of average football exposure. rCBF, a systems biology measure, was necessary for miRNA to affect behavior.

## Introduction

Athletes who participate in contact/collision sports are exposed to repeated head acceleration events (HAEs). HAEs can have long-term effects, regardless of etiology (i.e., athletics based vs. combat based) (Tagge et al. 2018). Three theories about the mechanism of injury from HAEs and concussion have been proposed: 1) “neurovascular decoupling” (Yuan et al. 1988; Yamakami and McIntosh 1989; Giza and Hovda 2001), 2) “neuroinflammation” (Aungst et al. 2014; Lozano et al. 2015; Chiu et al. 2016; Faden et al. 2016), and 3) “diffuse axonal injury” (Bazarian et al. 2007; Kumar et al. 2009; Hemphill et al. 2015; Mierzwa et al. 2015). Although these 3 models are independently supported by strong evidence, vascular function is a fundamental part of the initiation phase of inflammatory response, and its longer-term resolution phase (Xiong et al. 2018; Sankar et al. 2019). Thus, 2 of the 3 models have some overlap.

The neuroinflammation model was the focus of a recent study on microRNA (miRNA) levels before and over the course of the football season on a single NCAA Division I collegiate team (Papa et al. 2018). By comparing collegiate football players to nonathlete controls, Papas and colleagues found significantly elevated levels for a panel of miRNAs (miR-20a, miR-505, miR-92a, miR-195, miR-93p, miR-30d, miR-486, miR-362-3p, and miR-151-5p) previously reported in the literature to be associated with inflammatory responses to systemic disease and traumatic brain injury (TBI). Many of these miRNAs are known to be expressed in brain tissue (i.e., miR-20a, miR-30d, miR-92a, miR-195, and miR-151-5p) (Baraniskin et al. 2011; Schober et al. 2015; Lin et al. 2016; Liao et al. 2019; Wingo et al. 2020). The same panel of miRNAs has also been reported to be elevated in emergency room (ER) patients with TBI as contrasted with ER patients without TBI and was correlated with abnormal clinical readings on computed tomography scans (Bhomia et al. 2016). These prior findings suggest that neuroimaging may be able to localize brain perturbations resulting from systemic miRNA alterations following TBI.

MiRNAs are small (19–28 nt) endogenous RNA molecules that modulate gene expression by suppressing targeted messenger RNAs (mRNAs) (Fabian et al. 2010); they represent a dynamic measure of gene function and are part of the transcriptome (Bushati and Cohen 2007). Since each miRNA can target hundreds of mRNAs, miRNAs are involved in a wide variety of cellular processes, including many of those that occur after the initial physical impact in head injuries, such as the initiation of inflammation, or its longer-term resolution, both of which involve significant vascular control (Davidson-Moncada et al. 2010; Tahamtan et al. 2018). Emerging evidence suggests miRNAs may have diagnostic value for TBI, either for identifying mild TBI (mTBI) or distinguishing between mild and severe TBI (Di Pietro et al. 2017; Di Pietro et al. 2018). In the aforementioned study by Papa and colleagues, the level of the 9 miRNAs exhibited a negative relationship to Standard Assessment of Concussion (SAC) scores in the football athletes (Papa et al. 2018). Specifically, higher miRNA levels were associated with greater evidence of clinical symptoms (i.e., lower SAC scores) prior to the football season. These abnormalities occurred in players who had not had any head impacts in 12 weeks and were medically cleared to begin the upcoming season. In the context that the average history of competitive football play in these athletes was 11 years, these observations raised the hypothesis that the elevated levels were residual circulating miRNAs as a result of their continued participation in a contact sports, potentially reflecting chronic inflammation.

To better understand how these miRNAs contribute to the behavioral deficits, we used a multimodal approach to determine if this baseline miRNA abnormality impacted behaviors that are susceptible to HAEs/concussion and used magnetic resonance imaging (MRI) to localize these behavioral alterations to brain regions whose activity or perfusion is known to contribute to the behavioral process. The same panel of 9 miRNAs reported to be elevated in ER patients with TBI (Bhomia et al. 2016) and preseason football athletes (Papa et al. 2018) is a priori selected for this study due to their association with TBI and clinical symptoms. We focused on behavioral measures of motor control, using a validated virtual reality (VR) task grounded in the work of Luria (Slobounov and Sebastianelli 2014; Teel and Slobounov 2015).

The VR task produced computational behavior measures of 1) spatial navigation accuracy (“Spatial Memory”), 2) sensory–motor reactivity and efficiency of visual–spatial processing (“Reaction Time”), and 3) postural stability during equilibrium changes (“Balance”), along with 4) an integrative metric of all 3 measures together (“Comprehensive” score). Alexander Luria observed that head impacts without clinical signs of trauma may be associated with chronic neurocognitive deficits in 1) spatial orientation and accuracy of navigation, 2) processing of visual–spatial information (sensory–motor reactivity), or 3) related coordination functions such as whole-body postural control or balance (Luria 1948, 1962, 1973). He proposed that these deficits were due to patients’ inability to create a “cognitive map” of the perceptual-working space, making them prone to relying on short-term memory. He also noted patients could have problems reorienting themselves to generalized changes in the space around them and might have balance deficits (Slobounov and Sebastianelli 2014). Although a number of computer-based neuropsychological tests exist to assess aspects of Luria’s triad (i.e., [1]–[3] above), VR can detect residual abnormalities in the absence of self-reported symptoms by HAE recipients and long-term participants in impact sports (Teel et al. 2016a, 2016b).

Connecting such motor control abnormalities to regional perfusion in brain regions mediating motor control, such as the basal ganglia, primary sensorimotor cortex, premotor cortex, supplementary motor area, or cerebellum (DeLong and Strick 1974; Bostan and Strick 2018) would support the specificity of such observations. If this relationship between motor control abnormalities and regional brain perfusion could be linked to miRNA levels via a mediation analysis, it would represent a novel framework describing how circulating miRNA levels affect motor control through regional neuroimaging measures and link 3 distinct spatiotemporal scales of function (Braeutigam 2017). To date, no such relationships between miRNA levels, regional brain imaging, and computational behavior—what might be called “imaging omics”—have been reported in humans.

This study performed a 3-step analysis to test if a relationship existed between miRNA levels, regional neuroimaging measures, and behavior. Specifically, we tested a model where miRNA elevations observed before seasonal activities reflect residual elevations of inflammatory miRNAs related to abnormal regional perfusion in brain regions that produce behaviors affected by contact sport exposure and by HAEs. We used a candidate miRNA approach, focusing on the panel of 9 miRNAs reported to be elevated in the same athletes compared with age-matched, nonathlete controls by Papa et al. (2018). The analysis was carried out in 3 steps. First, we investigated if a 3-way set of associations existed between 1) miRNA level, 2) regional cerebral blood flow (rCBF) in motor control regions, and 3) computational behavior of motor control that did not depend on an intermediate phenotype like rCBF to connect miRNA levels to behavior. A whole-brain, voxelwise statistical approach was used for this exploratory step to determine regions where perfusion was related to either miRNA level or behavior. MiRNAs are a dynamic measure of systems function, and their relationship to behavior may go beyond the idea of an intermediate phenotype in “imaging genetics” (Meyer-Lindenberg and Weinberger 2006), wherein brain imaging acts as a common, overlapping node between 2 associations. Second, we used a directed mediation analysis, wherein miRNA levels were always the input variable between these 3 layers of spatiotemporal organization, and behavior was always the output variable. In such a framework, brain imaging is a required variable for the effect of miRNA on behavior and provides a proof-of-concept that the systems biology (measured by brain imaging) is needed for the effect of miRNA on behavior. Directed mediation thus requires confirmation of the absence of confounds such as miRNA levels mediating the relationship between rCBF and behavior and in such context has been argued to be mechanistic (Shrout and Bolger 2002) as opposed to purely associative (Spencer et al. 2005). Third, across any relationships of miRNA, rCBF, and behavior, we sought to determine if there were consistent patterns across all (rather than some or most of) the observed relationships. For illustration purposes, such consistent patterns might include a positive relationship between miRNA level and rCBF as one might expect with inflammatory processes. Other consistent patterns might include a negative relationship between rCBF and behavior, in line with other published results of football players (Talavage et al. 2014) or a negative relationship between miRNA level and behavior, parallel to what Papa et al*.* (2018) saw with miRNA and SAC scores. We used several methods to minimize false positives including corrections for multiple comparisons at each stage of analysis and graphical inspection for quality assurance of associations, along with a formal Cook’s Distance analysis of data point influence on results.

## Materials and Methods

### Subjects

A total of 24 collegiate male football athletes were recruited for this study. Written informed consent was obtained from all subjects, in accordance with the guidelines of the university’s institutional review board. Sample selection focused on 1) athletes who were starters for the team and would be anticipated to participate in all practices and games for the season, who also were 2) willing to complete all study procedures in 2 weeks prior to any contact practice at the beginning of the season and within 1 week of the last game. Analysis with G^*^Power based on imaging measures alone indicated that a sample of 24 subjects would produce power = 0.77, with the following parameters selected: effect size = 0.5, significance < 0.05, and power > 0.75. Demographics including age, years of play, football team position, and number of previous concussions diagnosed by medical personnel were collected based on self-report and confirmed by the team physician. Preseason MRI scans were collected within 1 week before the athletic season started. Only 23 subjects received the preseason MRI scan. Additional subjects were excluded due to missing miRNA or VR scores. For the final analysis, 20 subjects (age = 21 ± 2 years) were included for the preseason MRI, which yielded statistical power = 0.70 based on the same assumptions as stated above. The average number of years of participation in football was 11 ± 4 years, and 6 were speed players and 14 were nonspeed players based on classification criteria proposed by Lehman 2013. Two of the subjects had 2 prior concussions, 5 had 1 prior concussion, and the rest had no prior concussions.

### Serum Extraction

Blood samples were collected within a week before the season began. A total of 5 mL of blood was placed in a serum separator tube and centrifuged after clotting at room temperature. The serum was placed bar-coded aliquot containers and stored at −70°C until transport to a central laboratory for batch analysis. Personnel performing the laboratory analysis were blinded to the clinical data.

### VR Testing

During each visit, athletes also completed a previously validated VR neurocognitive testing with a 3D TV system (HeadRehab.com) and a head-mounted accelerometer. The test included 3 modules: spatial memory, balance, and whole-body reaction time. Scores from these modules were combined to generate a Comprehensive score.

Following the behavioral neurology of spatial memory problems in veterans with head injury described by Luria 1948, the spatial navigation task involved 3 modules. The first module, a memory test, showed subjects a randomized virtual pathway with multiple turns to a door along with the return trip. Subjects were instructed to repeat the pathway using a joystick, and their accuracy was assessed via correct responses versus errors. In the balance task, subjects were instructed to hold a tandem Romberg position for all trials. The virtual room was completely still for the first trial for a baseline measure. In the subsequent 6 trials, the virtual room moved in various directions, and individual alignment with the virtual room was quantified via a pressure plate on which they stood. For the reaction time module, subjects stood feet shoulder width apart with hands on their hips. They were instructed to move their body in the same direction as the virtual room’s movements, and the pressure plate measured response time latency. Raw data were run through mathematical algorithms to output scores on a scale of 0 (worst) to 10 (best). In addition to individual component scores, an overall “Comprehensive score” was calculated by combining the 3 test scores (Teel et al. 2016a, 2016b). These VR assessments were assessed at the same time as neuroimaging and serum extraction. Detailed descriptions of the modules and their sensitivity and specificity have been described previously (Teel et al. 2016a, 2016b).

### MRI Acquisition

Imaging data were collected on a 3.0 T whole-body Siemens Prisma scanner (Erlangen, Germany), equipped with a 32-channel head coil. For both pre- and postseason visits, high-resolution *T*_1_-weighted anatomical images and 3D-background suppressed (BS)-pulsed arterial spin labeling (PASL) images were acquired with the following parameters: 3D-magnetization-prepared rapid gradient echo–repetition time (TR)/echo time (TE)/inversion time (TI) = 1700/1.77/850 ms, flip angle = 9°, matrix size = 320× 260 × 176, voxel size = 1 mm isotropic, and parallel acceleration factor = 2 (total duration = 3 min 31 s); 3D-BS-PASL–PICORE/Q2TIPS labeling scheme, TR/TE = 4600/15.62 ms, matrix size = 128x128x120, voxel size = 1.5 mm × 1.5 mm × 3 mm, 40 axial slices, TI_1_/TI_2_ = 700/1990 ms, parallel acceleration factor = 2, 6 control/tag pairs, and 1 M0 acquisition (total duration = 6 min 2 s).

### CBF Mapping

Imaging data were processed using in-house scripts written in Matlab R2016a (Mathworks) with Statistical Parametric Mapping SPM8 (Wellcome Department of Imaging Neuroscience). All ASL data were motion corrected with the first image of the series as the reference and then coregistered to the high-resolution anatomical image. Perfusion-weighted images were generated by pairwise subtraction between control and tag images and averaged over the entire time series. Images were converted to quantitative CBF units in mL/100 g/min using the single-blood-compartment model (Wang et al. 2003):

$$ f=\frac{\lambda \cdot \Delta \mathrm{M}}{2\alpha \cdot{M}_0\cdot{\mathrm{TI}}_1\cdot{e}^{-{\mathrm{TI}}_2/{T}_{1b}}} $$

Where *f* represents CBF in quantitative units, ΔM is the perfusion-weighted signal, λ is the blood/water tissue partition coefficient (assumed to be 0.9 g/mL; Herscovitch and Raichle 1985), α is the inversion efficiency assumed to be 0.98 × 0.75 = 0.735 (Garcia et al. 2005), *M*_0_ is the equilibrium magnetization from the *M*_0_ acquisition, and *T*_1*b*_ is blood *T*_1_ assumed to be 1664 ms (Lu et al. 2004). The quantitative CBF maps were then transformed to Montreal Neurological Institute (MNI) template space and upsampled to 1.5-mm isotropic resolution based on transformation matrix calculated from the high-resolution anatomical image using VBM8 (Ashburner and Friston 2000). A 3D 3-mm smoothing kernel was applied to the spatially normalized CBF maps to minimize normalization discrepancies.

### miRNA Analysis

#### RNA Isolation

Serum samples from athletes were used to isolate total RNA. RNA was isolated using 100 lL of serum using a serum/plasma miRNA isolation kit (Qiagen Inc.) as per manufacturer’s recommended protocol. The RNA was eluted in 20 lL of DNAse–RNAse-free water and stored at −80C for further use. For a quality check of the RNA, a bioanalyzer assay using a small RNA assay was performed to confirm the quality of the RNA.

#### Droplet Digital PCR (ddPCR)

For absolute quantitation of miRNAs, we used a ddPCR platform (Bio Rad Inc.). For ddPCR reaction, 10 ng of total RNA was reverse transcribed using the specific miRNA TaqMan assays (Thermofisher Scientific Inc.) as per recommended protocol in a 15 μL total reaction volume; 5 μL of reverse-transcribed product was used to set up the real-time PCR reaction using miRNA TaqMan assays; and 20 μL of the final real-time PCR reaction was mixed with 70 μL of droplet oil in a droplet generator (Bio Rad Inc.). Following the droplet formation, the PCR reaction was performed as per recommended thermal cycling conditions. The final PCR product within the droplets was analyzed in a droplet reader (Bio-Rad Inc.). The total positive and negative droplets were measured, and the concentration of the specific miRNA/μL of the PCR reaction was determined. All the reactions were performed in duplicates.

### Statistical Analysis

Regression analyses were run to determine if history of concussion (HOC) and years of exposure (YoE) were correlated with either VR scores or miRNA levels. Since both variables were not significantly correlated with VR scores or miRNA levels, we report results below without incorporating HOC or YoE as covariates (data not shown). To supply hypotheses for future work, we also regressed HAEs measured over the following season with preseason measures of miRNA, rCBF, and VR behavior where cumulative and average HAE measures might be considered a crude estimate of prior seasonal exposure to impacts (see Supplementary Materials and Methods).

#### CBF and VR Scores Correlation

Statistical Parametric Mapping (SPM8, Wellcome Trust Centre for Neuroimaging, UK) was used to calculate voxelwise statistics on the preseason CBF maps. Each of the VR scores was entered as a covariate in a 1-sampled *t*-test, and *T*-contrasts for positive and negative correlations between preseason CBF and VR score were used to locate areas with significant correlations. To correct for multiple comparisons, all whole-brain statistics were determined by combining a voxelwise *P* value of 0.005 with the cluster size calculated using AFNI’s 3dClustSim command with mixed autocorrelation function model (Cox et al. 2017) to reach a corrected *P* value of 0.05 (Version AFNI_19.2.23). The cluster threshold for this analysis was 203 voxels. Anatomical localization of all clusters was determined based on the Harvard–Oxford cortical (48 regions) and subcortical (21 regions) atlases (Desikan et al. 2006) and a probabilistic cerebellar atlas with 28 anatomical regions (Diedrichsen et al. 2009). The percentages listed in tables represent the percentage of each anatomical region occupied by the cluster.

#### CBF and miRNA Levels Correlation

The 3DClustSim approach as described above was used to determine the correlation between preseason CBF and each of the preseason miRNA levels. The cluster threshold for each miRNA analysis to reach a cluster-level corrected *P* = 0.05 is listed in Table 2.

#### Influence Testing (Cook’s Distance Computation)

Before data were submitted for 3-way association (below), using the outcomes of the CBF and VR analysis and the CBF and miRNA levels analysis, we performed an influence assessment to rule out associations being driven by a minority of the data. Using the program STATA, the Cook’s Distance method was used to determine significant outlier effects on regression analyses. First, miRNA values were regressed against MRI data (maxvox and cluster). Any Cook’s values (*D*) >5 signifies a likely significant outlier effect on regression results. Regression analyses of miR-505 and miR-486 against mean cluster rCBF and maxvox exhibited *D* > 5 (miR-505—cluster1 cluster/maxvox = 5.7/1.54, cluster2 cluster/maxvox = 5.94/2.48; miR-486—cluster1 cluster/maxvox = 14.8/13.9, cluster2 cluster/maxvox = 16.5/12.4). These outliers were removed from the data set and regressions were rerun. After removal of these outliers, the regressions were no longer significant. Balance data were also regressed against miRNA, maxvox, and cluster; no regressions resulted in *D* > 5.

#### CBF, miRNA, and VR Scores Interaction

This analysis evaluated 3-way associations across these 3 measures. To pinpoint regions where there were significant correlations between CBF and miRNA and CBF and VR scores, the CBF and VR score correlation results were used as a mask for small volume correction in the CBF and miRNA correlation analysis, which was thresholded at *P* < 0.005 uncorrected and clusters >20 voxels. The small volume correction performs multiple comparisons correction only within voxels that had a significant CBF and miRNA correlation. Clusters that survive familywise error (FWE) correction at *P(FWE)* < 0.05 were reported. To test the robustness of our results, we also reversed the direction of the analysis by using the CBF and miRNA results as mask for the CBF and VR scores correlations. The same clusters were identified with slightly different *T* values and location of voxel with maximum *T* value. Therefore, only the VR score-masked CBF and miRNA correlation results were reported.

#### Mediation Analysis

Clusters that had significant CBF and miRNA and CBF and VR score correlations were used as input for the mediation analysis (Hayes 2009), which tested whether the effect of the independent variable (IV) on the dependent variable (DV) was transmitted by the mediator (M) (MacKinnon et al. 2007; Hayes 2009). Mediation analysis used a standard stepwise process:

$$\mathrm{Step}\ \mathrm{A}:\mathrm{DV}={\beta}_0+{\beta}_{1A}\mathrm{IV}+\epsilon $$

$$\mathrm{Step}\ \mathrm{B}:\mathrm{DV}={\beta}_0+{\beta}_{2B}M+\epsilon $$

$$\mathrm{Step}\ \mathrm{C}:\mathrm{DV}={\beta}_0+{\beta}_{1C}\mathrm{IV}+{\beta}_{2C}M+\epsilon $$

Step D: Use Sobel’s test to check if ${\beta}_{1C}$ is significantly lower than ${\beta}_{1A}$. If ${P}_{\mathrm{Sobel}}<0.05$, the mediation is significant. Sobel tests that met a Bonferroni correction for all tests performed were also noted.

To test the directed mediation, the miRNA level was entered as the IV, with either the cluster averaged CBF value (mean cluster) or the CBF value extracted from the voxel with maximum *T* value (Maxvox) as M, and VR Balance score as the DV. To confirm the mediation effects, we also tested a control mediation where the cluster CBF value was entered as the IV and the miRNA level as M. We expected the Sobel’s test *P* value for all control mediations to be >0.05.

## Results

Analysis involved 5 steps, with graph evaluation and Cook’s Distance analyses—a gold standard engineering approach—performed after inductive statistics to assess effects of potential outlier data (Cook 1977). We first performed analyses of 2-way interactions: 1) between rCBF and VR scores and 2) between rCBF and miRNA levels, followed by analysis of 3-way interactions between rCBF, VR, and miRNA to quantify potential overlap between the results of 1) and 2). The 3-way analyses were done in 2 ways: 1) using the rCBF–VR results as a mask for rCBF–miRNA data and 2) using the rCBF–miRNA results as a mask for rCBF–VR data to assess consistency of results. We then identified 4 miRNAs showing nominal effects in 2-way association with VR measures and performed 5 directed mediation analyses between rCBF, miRNA, and VR measures. For reporting, directed mediation analyses were required to show Sobel *P* values < 0.05 across “both” cluster and voxel with maximum *T* statistics (maxvox) signals, with percent mediation greater than that of the control mediation in both. Further, control mediation analyses (where miRNA was the mediator and rCBF the IV) were required to be nonsignificant (all Sobel *P* > 0.05) for both maxvox and cluster data.

### rCBF and VR Scores Correlation

Two-way analysis between rCBF and VR scores revealed negative correlations between rCBF and 2 of the 4 VR measures: Comprehensive score and Balance score. A negative correlation means better performance (higher VR score) was associated with lower rCBF. Although the Balance score contributed to the Comprehensive score, the clusters detected for these 2 values were in different brain regions, suggesting that the Comprehensive score results were not solely driven by the Balance scores. Comprehensive score was negatively correlated with rCBF in the thalamus, post cingulate/precuneus, and lateral temporal areas. The Balance score, on the other hand, was correlated with rCBF in several regions including the bilateral putamen and in fronto-orbital cortex (FOC). A detailed summary of the location and size of clusters is shown in Table 1. The clusters, overlaid onto a single subject’s anatomical image in MNI space, and corresponding scatterplots are shown in Supplementary Figure 1*a*) results for Comprehensive score, and Supplementary Figure 1*b*) results for Balance score.

**Table 1:** Summary of clusters with significant correlations between CBF and VR scores

	Dir	*N*_voxels_	Peak *T*	*P*(unc)	*x*, *y*, *z* (mm)	Region	Label
Comprehensive	+ve	n.s.					
	−ve	285	8.16	6.23E-08	54, −33, 34	Parietal	R POC (37%), R PT (18%), R anterior SMG (8%), R anterior SMG (8%), R posterior SMG (2%)
		299	7.46	2.31E-07	−8, −41, 34	Limbic	L posterior cingulate (44%), L precuneus (39%)
		265	5.53	1.22E-05	3, −2, 4	Sublobar	L thalamus (53%), R thalamus (25%)
		206	5.05	3.54E-05	45, −39, 5	Temporal	R posterior SMG (35%), R tempero-occipital MTG (12%), R posterior STG (3%)
		210	4.82	5.97E-05	−47, −62, 33	Parietal	L superior LOC (83%), L angular gyrus (18%)
Spatial memory	+ve −ve	n.s.					
Balance	+ve	n.s.					
	−ve	999	6.93	6.59E-07	−18, 30, −20	Frontal	L FOC (23%), L subcallosal (9%), L putamen (20%), L subcallosal (9%), L FMC (4%), L frontal pole (2%), L insula (2%)
		1219	5.93	5.25E-06	27, 6, −8	Sublobar	R putamen (34%), R FOC (11%), R subcallosal (9%), R caudate (8%), R amygdala (5%), R pallidum (3%), R accumbens (3%), R FMC (3%), R frontal pole (2%)
		388	5.78	7.24E-06	66, −21, −1	Temporal	R posterior STG (75%), R anterior STG (7%), R posterior MTG (4%), R anterior MTG (2%)
		217	5.15	2.84E-05	−5, 9, −11	Frontal	L caudate (35%), L accumbens (34%)
Reaction time	+ve	n.s.					
	−ve						

Note: The peak *T* value within the cluster and its location are included in this table. Percentage values in the label column represent the percentage of anatomical region defined on the Harvard–Oxford atlas occupied by the cluster. Dir = direction, *N*_voxels_ = number of voxels in cluster, R = right, L = left, POC = parietal operculum cortex, PT = planum temporale, SMG = supramarginal gyrus, MTG = middle temporal gyrus, STG = superior temporal gyrus, LOC = lateral occipital cortex, FMC = frontal medial cortex, FOC = fronto-orbital cortex, n.s. = nonsignificant. Uncorrected is abbreviated as “unc”.

### rCBF and miRNA Correlation

Of the 9 miRNAs tested for 2-way relationship with rCBF, 2 showed no significant correlations: miR-362-3p and miR-93p. Examination of the correlation plots between rCBF and miRNA levels revealed potential outliers for miR-505 and miR-486, which was confirmed by Cook’s Distance analysis. Therefore, results for these 2 miRNAs are in Supplementary Table 1 and excluded from further analysis. Of the remaining miRNAs that had significant correlations, miR-20a, miR-30d, and miR-151-5p had positive correlations, where higher rCBF was associated with higher miRNA levels. For miR-20a, the significant clusters were located in multiple brain regions, including the brainstem and right cerebellum, FOC, caudate, putamen, and thalamus. Similar clusters were found for miR-30d. MiR-151-5p results were limited to the caudate and thalamus. The remaining miRNAs, miR-92a and miR-195, showed both positive and negative correlations with rCBF. Interestingly, positive correlations between rCBF and miR-20a, miR-92a, and miR-30d colocalized to the same clusters involving regions in the basal ganglia and FOC. Table 2 and Supplementary Figure 2 summarize the location and size of these clusters.

**Table 2:** Clusters with significant correlations between preseason CBF and miRNA levels

miRNA	*N*_voxels_	Peak *T*	*P*(unc)	*x*, *y*, *z* (mm)	Region	Label
miR-20a (*k* = 208)	434	6.62	2.17E-06	27, −15, 5	Sublobar	R putamen (46%), R pallidum (46%)
	230	6.62	2.19E-06	0, −30, −12	Midbrain	Brainstem (90%), R cerebellum I-IV (9%)
	855	5.93	8.27E-06	−18, 23, −20	Frontal	L FOC (30%), L subcallosal (13%), L putamen (6%), L paracingulate (4%)
	316	5.80	1.07E-05	−17, 2, 9	Sublobar	L thalamus (35%), L caudate (17%), L pallidum (4%)
	645	5.74	1.21E-05	27, 20, 3	Sublobar	R FOC (27%), R putamen (18%), R frontal pole (10%), R subcallosal (9%), R accumbens (3%), R caudate (2%)
	214	5.59	1.63E-05	−48, −17, 21	Parietal	L COG (50%)
miR-30d (*k* = 199)	324	7.2	5.16E-07	−41, −6, 18	Sublobar	L COG (84%), L insula (9%), L frontal operculum. (4%), L pars opercularis (1%)
	1752	7.2	5.54E-07	27, 11, −5	Sublobar	R putamen (32%), R subcallosal (4%), R COG (15%), R FOC (7%), R pallidum (5%), R subcallosal (4%), R insula (3%), R caudate (3%), R accumbens (2%), R frontal operculum (2%)
	364	5.8	8.44E-06	−23, 17, −1	Sublobar	L putamen (42%), L FOC (19%)
	230	5.2	2.74E-05	−18, −9, 15	Sublobar	L thalamus (84%)
miR-92a (*k* = 208)	258	5.5	1.44E-05	27, −15, 5	Sublobar	R putamen (48%), R pallidum (19%)
	325	5.5	1.56E-05	33, 12, 15	Sublobar	R putamen (33%), R COG (11%), R insula (10%), R frontal operculum (4%)
	287	5.1	4.09E-05	−18, 23, −21	Frontal	L FOC (41%), L putamen (27%)
	221	−7.9	0.000566	−21, −55, −18	Post. cerebellum	L cerebellum V (48%), L cerebellum VI (44%), L lingual (5%), L tempero-occipital fusiform (2%)
miR-195 (*k* = 204)	246	6.4	2.64E-06	−62, −13, 15	Parietal	L COG (61%), L postcentral (16%), L POC (5%), L Heschl’s (5%)
	244	−8.62	4.19E-08	0, 48, −15	Frontal	R FMC (41%), L FMC (38%), R frontal pole (16%), L frontal pole (6%)
	219	−5.77	8.99E-06	12, −57, 6	Limbic	R lingual (58%), R precuneus (37%), R intracalcarine (6%)
miR-151-5p (*k* = 193)	316	7.1	6.54E-07	15, −1, 13	Sublobar	L thalamus (32%), R thalamus (15%), R caudate (7%), R caudate (7%)

Note: Negative correlations are denoted by a minus sign before the peak *T* value. COG = central opercular cortex.

### rCBF, VR Scores, and miRNA Interaction

**Figure 1:**
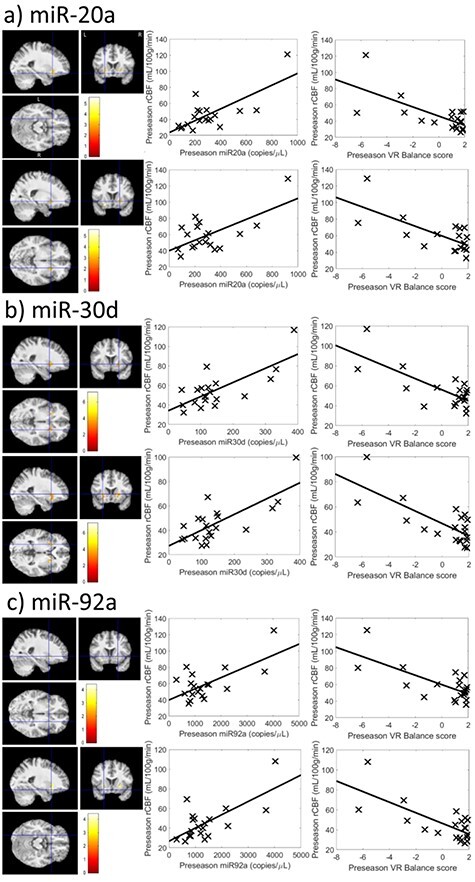
Clusters where significant correlations between CBF and miRNA levels and VR Balance scores were detected and their corresponding scatterplots. (*a*) miR-20a results, (*b*) miR-30d results, and (*c*) miR-92a results. Results for miR-505^*^ and miR-486 were excluded as they did not survive Cook’s Distance analysis.

Analyses using 1) rCBF–VR Balance score results as a mask for the rCBF–miRNA correlations largely overlapped those using 2) rCBF–miRNA correlations as a mask for the rCBF–VR Balance score correlations. Therefore, only the results for (1) are tabulated in Table 3 and shown in Figure 1.

**Table 3:** Summary of clusters with significant 3-way interactions between preseason CBF, miRNA levels, and VR Balance score

miRNA	Cluster *P*(FWE)	*N*_voxels_	Peak *T*	*x*, *y*, *z* (mm)	Region	Label
miR-20a	0.002	223	5.55	−18, 24, −20	Frontal	L FOC (36%), L putamen (11%), L subcallosal (3%)
	0.018	122	4.05	21, 14, −11	Sublobar	R putamen (77%), R accumbens (7%), R caudate (6%)
miR-30d	6.75E-06	503	7.18	27, 11, −5	Sublobar	R putamen (45%), R FOC (18%), R caudate (6%), R subcallosal (6%), R accumbens (3%)
	0.0004	287	5.80	−23, 17, 0	Sublobar	L putamen (40%), L FOC (15%)
miR-92a	0.043	93	4.41	27, 11, −5	Sublobar	R putamen (85%)
	0.004	184	4.37	−32, 5, 1	Sublobar	L putamen (35%), L FOC (21%)
miR-195	n.s.					
miR-151-5p						

Note: FWE means familywise error.

Three of the 7 tested miRNAs showed 3-way relationships with preseason rCBF and VR Balance score: miR-20a, miR-30d, and miR-92a. These findings colocalized to 2 clusters: 1) extending from the left posterior FOC to the left putamen (Put) and insula (INS) and 2) in the right Put. Summary data for these clusters are listed in Table 3 and their corresponding locations in MNI space and scatterplots are shown in Figure 1. Visualization-based localization, using published neuroanatomic landmarks (Caviness et al. 1996), was performed by a motor control and basal ganglia expert (A.J.B.) to check and complement the automated localization for these clusters and determined that the left rCBF focus extended from anterior Put to anterior INS/claustrum to subcallosal cingulate/ventromedial prefrontal cortex and posterior FOC (with some variability across the 3 miRNAs), whereas the right rCBF focus was localized primarily within the anterior Put, with extension to caudate and/or nucleus accumbens in some cases.

No significant clusters were detected when the rCBF–VR Comprehensive score results were used as a mask for the rCBF–miRNA correlations.

### miRNA and VR Scores Correlation

Table 4 shows the Pearson correlation *r* and *P* values for pairwise correlations between preseason miRNA levels and VR scores. Only VR Comprehensive and Balance scores are shown as these were the only scores significantly correlated with CBF in the image-based correlation analysis. Both scores were correlated (*P* < 0.05) with levels of miR-20a, miR-505, miR-30d, miR-92a, miR-486, and miR-151-5p. Additionally, a trend effect was observed for miR-195 levels and both VR Comprehensive and Balance scores (*P* ~ 0.07). All of these correlations were negative, where higher levels of miRNA were associated with worse VR performance.

**Table 4:** Associations between VR scores and miRNA levels

Behavioral measure	Statistics	miRNA								
		20a	505	362_3p	30d	92a	486	195	9_3p	151_5p
Comprehensive	Pearson *r*	**−0.538**	**−0.471**	−0.079	**−0.506**	**−0.444**	**−0.445**	*−0.406*	−0.332	**−0.544**
	*P* value	**0.014**	**0.031**	0.728	**0.016**	**0.039**	**0.038**	*0.061*	0.131	**0.009**
	*n*	20	21	22	22	22	22	22	22	22
Balance	Pearson *r*	**−0.460**	**−0.693**	−0.084	**−0.581**	**−0.463**	**−0.503**	*−0.396*	−0.230	**−0.634**
	*P* value	**0.041**	0.000^*^^*^	0.711	0.005^*^^*^	**0.030**	**0.017**	*0.068*	0.303	0.002^*^^*^
	*n*	20	21	22	22	22	22	22	22	22

Note: Bold indicates a nominally significant association at the *P* < 0.05 level, and italics indicate a trend effect. ^*^^*^ indicates results were significant with a Bonferroni correction (i.e., *P* = 0.05/9 miRNAs = 0.006); Comprehensive results are presented to guide future work given the 3-way analyses showed no significant effects for miRNA–CBF–VR Comprehensive associations.

### Mediation Results

Mediation analysis focused on the 2 rCBF foci (left Put-INS-FOC and right Put) identified for 3 miRNAs (miR-20a, miR-30d, miR-92a) from the analysis of interaction between rCBF, VR Balance scores, and miRNA. rCBF values extracted from both the maxvox and entire cluster were used for 1) directed mediation, (i.e., miRNA predicting rCBF and rCBF predicting Balance) and 2) control mediation, (i.e., rCBF predicting miRNA, miRNA predicting balance). All results are shown in Table 5. Given the number of statistical tests run for the mediation analyses, stringent criteria were used to determine the significance of the mediation analysis results. Mediation was considered to be significant only if 1) the Sobel’s test *P* value <0.05 for the mean cluster (a larger volume of tissue than just a maxvox), and both the mean cluster and maxvox had a directed mediation effect greater than that of the control mediation. Alternately, 2) if any Sobel test *P* value met the Bonferroni correction for multiple comparisons, it was considered significant.

**Table 5:** Mediation results

Model 1: IV Path A → M Path B → DV, Model 2: IV Path C → DV				Path A: IV predicting mediator		Path B: mediator predicting DV		Path C: IV predicting DV (model 1)		Path C: IV (with mediator) predicting DV (model 2)		Effect mediated	Sobel test
		**IV**	**M**	**Std β**	***P***	**Std β**	**p**	**Std β**	***P***	**Std β**	***P***	**%**	***P***
R Put (21, 14, −11)	Directed	miR-20a	MaxVox	0.701	0.001	−0.571	0.011	−0.469	0.043	−0.135	0.644	71	0.123
		miR-20a	Mean cluster	0.643	0.003	−0.700	<0.001	−0.469	0.043	−0.031	0.894	93^*^	0.026^*^
	Control	MaxVox	miR-20a	0.701	<0.001	−0.469	0.043	−0.571	0.011	−0.476	0.115	17	>0.20
		Mean cluster	miR-20a	0.643	0.003	−0.469	0.043	−0.700	<0.001	−0.679	0.010	3^*^	>0.20^*^
L Put-INS-FOC (−18, 24, −20)	Directed	miR-20a	MaxVox	0.803	<0.001	−0.553	0.014	−0.469	0.043	−0.071	0.842	85	0.169
		miR-20a	Mean cluster	0.736	<0.001	−0.685	0.001	−0.469	0.043	0.078	0.775	85	0.169
	Control	MaxVox	miR-20a	0.803	<0.001	−0.469	0.043	−0.553	0.014	−0.496	0.174	10	>0.20
		Mean cluster	miR-20a	0.736	<0.001	−0.469	0.043	−0.685	0.001	−0.743	0.014	0	>0.20
R Put (27,11,-5)	Directed	miR-30d	MaxVox	0.861	<0.001	−0.589	0.006	−0.614	0.004	−0.416	0.279	32	>0.20
		miR-30d	Mean cluster	0.737	<0.001	−0.744	<0.001	−0.614	.004	−0.145	0.550	76	0.020
	Control	MaxVox	miR-30d	0.861	<0.001	−0.614	0.004	−0.589	.006	−0.230	0.544	61	>0.20
		Mean cluster	miR-30d	0.737	<0.001	−0.614	0.004	−0.744	<0.001	−0.638	0.015	14	>0.20
**L Put-INS-FOC (−23,17, 0)**	**Directed**	**miR-30d**	**MaxVox**	**0.807**	**<0.001**	**−0.807**	**<0.001**	**−0.614**	**0.004**	**0.106**	**0.666**	**100**	**0.002**
		**miR-30d**	**Mean cluster**	**0.737**	**<0.001**	**−0.740**	**<0.001**	**−0.614**	**0.004**	**−0.151**	**0.536**	**75**	**0.022**
	**Control**	**MaxVox**	**miR-30d**	**0.807**	**<0.001**	**−0.614**	**0.004**	**−0.807**	**0.000**	**−0.893**	**0.002**	**0**	**>0.20**
		**Mean cluster**	**miR-30d**	**0.737**	**<0.001**	**−0.614**	**.004**	**−0.740**	**<0.001**	**−0.629**	**0.017**	**15**	**>0.20**
**R Put (27,11,-5)**	**Directed**	**miR-92a**	**MaxVox**	**0.720**	**<.001**	**−0.589**	**0.006**	**−0.465**	**0.039**	**−0.085**	**0.768**	**82**	**0.085**
		**miR-92a**	**Mean cluster**	**0.647**	**0.002**	**−0.702**	**<0.001**	**−0.465**	**0.039**	**−0.018**	**0.938**	**96**	**0.020**
	**Control**	**MaxVox**	**miR-92a**	**0.720**	**<0.001**	**−0.465**	**0.039**	**−0.589**	**.006**	**−0.528**	**0.079**	**10**	**>0.20**
		**Mean cluster**	**miR-92a**	**0.647**	**0.002**	**−0.047**	**0.039**	**−0.702**	**<0.001**	**−0.691**	**0.007**	**2**	**>0.20**
L Put-INS-FOC (−32,5,1)	Directed	miR-92a	MaxVox	0.717	<0.001	−0.526	0.017	−0.465	0.039	−0.180	0.546	61	0.196
		miR-92a	Mean cluster	0.691	<0.001	−0.722	<0.001	−0.465	0.039	0.066	0.780	100^*^	0.010^*^
	Control	MaxVox	miR-92a	0.717	<0.001	−0.465	0.039	−0.526	0.017	−0.396	0.194	25	>0.20
		Mean cluster	miR-92a	0.691	<0.001	−0.047	0.039	−0.722	<0.001	−0.768	0.004	0^*^	>0.20^*^

Note: rCBF from either the voxel with the maximum *T*-value (maxvox) or mean over the entire cluster (mean cluster) was used in these analyses. Results were considered significant if 1) both maxvox and mean cluster show a directed mediation effect greater than the control mediation effect, 2) directed mediation Sobel’s test *P* value < 0.05, and 2) control mediation Sobel’s test *P* value >0.05. Any mediation meeting a Bonferroni correction for multiple comparisions (*P* < 0.002) was also noted. Both the Left Put-INS-FOC cluster of miR-30d and right Put cluster of miR-92a satisfy these criteria and are shown in bold. Results where only maxvox or mean cluster but not both satisfy the above criteria are marked with ^*^, these include the right Put cluster for miR-20a, R Put-INS-FOC cluster for miR-30d, and Left Put-INS-FOC cluster for miR-92a.

**Figure 2:**
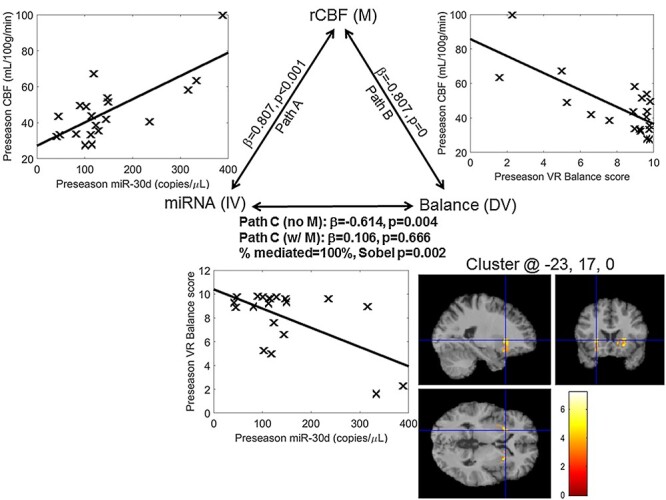
Mediation results for miR-30d, extracted from the L Put-INS-OFC cluster. Scatterplots for each of the 3 pairwise correlations are shown, as well as the corresponding beta and *P* values. The cluster from which rCBF values were extracted for these plots is shown in the bottom right, overlaid onto a single subject’s anatomical image.

Based on the above criteria, the mediation analyses revealed 3 general outcomes. First, 2 significant results were observed for 1) the right Put, miR-92a, and Balance score and 2) the left Put-INS-FOC, miR-30d, and Balance score. The average percent mediation effect across both maxvox and cluster analyses was 88% and 89%, respectively, compared with an average of 6% and 8% for the control mediations. These results are shown in bold in Table 5. Scatterplots for each of the pairwise correlations relevant for these results, as well as the location of the clusters overlaid onto a high-resolution anatomical image in MNI template space, are shown in Figure 2. Notably, the left Put-INS-FOC maxvox for miR-30d was the strongest result, with a *P* value of *P* = 0.002, meeting Bonferroni correction for all Sobel tests computed (12 directed mediation and 12 control mediations or *P* < 0.05/24 = 0.0021). The maxvox rCBF in this case mediates 100% of the relationship between miR-30d levels and VR Balance, whereas the control mediation was 0%. Second, trend effects were noted for 3) the right Put, miR-20a, and Balance score and 4) the left Put-INS-FOC, miR-92a, and Balance score. These results were considered trend effects as only the mean cluster rCBF satisfied the criteria for significance described above. They are denoted by ^*^ in Table 5. Third, nonsignificant or inconsistent effects were noted for 5) the right Put, miR-30d, and Balance score, and 6) the left Put-INS-FOC, miR-20a, and Balance score, where either none of the directed mediation results were significant (6) or the percent mediation was higher in the control mediation case (5). A detailed description of the most robust findings (i.e., [1] and [2]) is included below.

#### miR-30d, Left Put-INS-FOC, and VR Balance

Pairwise regressions between 1) miR-30d and cluster rCBF in the left Put-INS-FOC (β = 0.737, *P* < 0.001), 2) cluster rCBF and VR Balance score (β = −0.740, *P* < 0.001), and 3) miR-30d and VR Balance score (β = −0.614, *P* = 0.004) were all significant ignoring the mediator (cluster rCBF). After accounting for the effect of the mediator, the relationship between miR-30d and VR Balance score was no longer significant (β = −0.151, *P* > 0.2), with the mediator explaining 75%, and Sobel *P* = 0.022. Control mediation analysis, wherein miRNA was the mediator, showed 15% effect mediated, and Sobel *P* > 0.2.

For this 3-way relationship and mediation, there was also a salient result for the maxvox of the left Put-INS-FOC*.* Specifically, pairwise regressions between 1) miR-30d and maxvox rCBF in the left Put-INS-FOC (β = 0.807, *P* < 0.001), 2) maxvox rCBF and VR Balance score (β = −0.807, *P* < 0.001), and 3) miR-30d and VR Balance score (β = −0.614, *P* = 0.004) were all significant ignoring the mediator (maxvox rCBF). After accounting for the effect of the mediator, the relationship between miR-30d and VR Balance score was no longer significant (β = 0.106, *P* > 0.2), with the mediator explaining 100%, and Sobel *P* = 0.002 (meeting a Bonferroni correction for all mediation analyses run). Control mediation analysis, wherein miRNA was the mediator, showed 0% effect mediated, and Sobel *P* > 0.2.

#### miR-92a, Right Put, and VR Balance

Pairwise regressions between 1) miR-92a and cluster rCBF in the right Put (β = 0.647, *P* = 0.002), 2) cluster rCBF and VR Balance score (β = −0.702, *P* < 0.001), and (3) miR-92a and VR Balance score (β = −0.465, *P* = 0.039) were all significant ignoring the mediator (cluster rCBF). After accounting for the effect of the mediator, the relationship between miR-30d and VR Balance score was no longer significant (β = −0.018, *P* > 0.2), with the mediator explaining 96%, and Sobel *P* = 0.02. Control mediation analysis, wherein miRNA was the mediator, showed 2% effect mediated, and Sobel *P* > 0.2.

For this 3-way relationship and mediation, there was also a trend effect for the maxvox of right Put*.* Specifically, pairwise regressions between 1) miR-92a and maxvox rCBF in the right Put (β = 0.720, *P* < 0.001), 2) maxvox rCBF and VR Balance score (β = −0.589, *P* = 0.006), and 3) miR-92a and VR Balance score (β = −0.465, *P* = 0.039) were all significant ignoring the mediator (maxvox rCBF). After accounting for the effect of the mediator, the relationship between miR-92a and VR Balance score was no longer significant (β = −0.085, *P* > 0.2), with the mediator explaining 82%, and Sobel *P* = 0.085. Control mediation analysis, wherein miRNA was the mediator, showed 10% effect mediated, and Sobel *P* > 0.2.

## Post Hoc Analyses for Hypothesis Generation

We regressed HAEs measured over the following season with preseason measures of miRNA and rCBF (using data from the mediation analyses in Table 5), with the assumption that cumulative and average HAE measures might have some similarity to prior seasonal exposure to impacts. These exploratory results showed nominal effects (*P* < 0.05, uncorrected) for 6 of the 9 miRNA’s (miR-20a, miR-505, miR-362-3p, miR-30d, miR-92a, miR-195, miR-9-3p) (data not shown). Two rCBF measures showed nominal regressions (*P* < 0.05, uncorrected), including a rCBF focus in the putamen that mediated 82% of the effects for miR-92a (data not shown). No behavior measures were associated with HAEs (all *P* > 0.05).

## Discussion

This study examined whether a previously identified panel of miRNAs distinctly elevated among football players exposed to a long-term impact play (Papa et al. 2018) was associated with rCBF and postural control measures, and if rCBF mediated the effects of miRNA on behavior. We hypothesized that elevated miRNA levels observed before seasonal play for collegiate football players were potentially residual signs of inflammation as supported by the presence of a 3-way interaction and specified direction to the associations, between preseason miRNA levels, rCBF of brain regions producing motor control behavior, and motor control measured by sensitive VR tasks. Our study supported this model with a number of findings: 1) rCBF was negatively correlated with VR Balance and Comprehensive scores (i.e., elevated rCBF was associated with worsened VR performance) uniformly in task-relevant regions, such as the left Put-INS-FOC and right Put. 2) rCBF was differentially correlated with a subset of the miRNAs tested, and where there was a 3-way relationship between miRNA, rCBF, and VR behavior, it was always positively associated with miRNA levels. (3) miRNA levels and VR behavior showed only negative associations, so that higher miRNA levels were associated with worsening behavior. (4) For the 3-way interactions observed, rCBF consistently mediated the detrimental effects of miRNAs on VR Balance, and there was no partial mediation with miRNA mediating the association of rCBF with VR behavior.

Mediation analyses specifically focused on 3 miRNAs and 2 basal ganglia loci involving the bilateral Put (Table 5). Two of these 3 analyses were quite robust—one involving miR-30d and the left Put-INS-FOC, and the other involving miR-92a and the right Put—and should be replicable in other HAE athlete samples. Two other results were suggestive but will likely require larger samples for retesting as the cluster and maxvox results did not agree—these included miR-20a and the right Put, and miR-92a and the left Put-INS-FOC. Lastly, 2 of these results showed inconsistent findings between maxvox and mean cluster data (miR-30d and right Put-INS-FOC) or high Sobel *P*-values (>0.05) for maxvox and mean cluster data (miR-20a and left Put); larger sample sizes are likely to produce different results from those reported herein. Notably, the directionality of the 3-way relationships observed was consistent across all 6 analyses and support the following hypothesized model: 1) Participation in contact sports such as football induces a persistent neuroinflammatory response as evidenced by elevated levels of select miRNAs; 2) the putamen, thought to be fundamental in motor control processes such as balance/postural stability (Horak et al. 1992; Bloem et al. 1995; Horak et al. 2005; Blood 2013), is affected by sports participation and hypothesized chronic inflammation; 3) chronic putaminal inflammation is associated with impaired motor control behavior.

### Better VR Performance Is Associated With Lower rCBF in Task-Relevant Brain Regions

Negative correlations were found between rCBF and VR Comprehensive and Balance scores in all our analyses. For the VR Comprehensive score, rCBF from brain regions such as the thalamus, posterior cingulate, and bilateral supramarginal gyrus were negatively associated with VR score. The posterior cingulate and the adjacent retrosplenial cortex have long been found to be associated with spatial orientation and working memory, as is evident from both animal and human lesion models (Vogt et al. 1992; Maguire 2001). Primate studies have shown that these regions receive afferents from the anterior thalamus (Vogt et al. 1987), an area also revealed to be significantly correlated to VR Comprehensive score in the current study. The correlations between rCBF in these regions and VR Comprehensive score are likely driven by the spatial memory and orientation (Reaction Time) components of the VR task. On the other hand, the VR Balance score was associated with brain perfusion magnitude in the putamen, which is thought to be involved in postural and balance control, in addition to other aspects of motor function (Horak et al*.* 1992; Bloem et al*.* 1995; Horak et al*.* 2005; Blood 2013). It is particularly interesting that the Balance score was not associated with perfusion in the cerebellum, given its more established role in balance function relative to the basal ganglia. Blood (2013) hypothesized that the basal ganglia, and particularly the putamen, coordinate(s) different aspects of balance and postural control across many different states and contexts by coordinating recruitment of programs across multiple motor regions (including the cerebellum). It may be that adaptive, dynamic postural responses such as those required by the VR task are linked most tightly to function in the putamen, even if other regions involving balance function (such as the cerebellum) are also impacted by HAEs. Interestingly, only the VR Balance score was concurrently related to rCBF and miRNA levels, with strong mediation results.

### MicroRNA Accumulation Is Detrimental to VR Performance and May Be Related to Neuroinflammation

In a previous study on the same cohort, Papa et al. (2018) reported that the preseason miRNA levels in these football athletes were significantly elevated compared to healthy young nonathlete controls (Papa et al. 2018). Accumulation of these miRNAs appeared to be detrimental to the athletes’ performance on cognitive tasks, as Papa et al. (2018) also reported that athletes with higher SAC scores (i.e., better neurocognitive performance) had lower miRNA levels. This is corroborated in our correlation analysis, which revealed a negative correlation between miRNA levels and VR performance.

Of the 3 miRNAs that emerged with a significant 3-way interaction with rCBF and VR Balance score, miR-20a and miR-92a are part of the miR-17-92 cluster on chromosome 13 (Concepcion et al. 2012). MiRNA clusters are comprised of multiple miRNAs that are transcribed by physically adjacent sequences and often target a common factor or signaling pathway. The miR-17-92 cluster has a crucial role in the immune system, as mice deficient in this cluster die shortly after birth and overexpression of this cluster leads to lymphoproliferative disease and autoimmune disorders (see Concepcion et al*.* 2012 for review). MiR-20a, in particular, has been shown to inhibit the secretion of cytokines such as IL-6, IL-8, and IL-10 (Reddycherla et al. 2015), indicative of its involvement in neuroinflammatory processes. In a murine model of traumatic spinal cord injury, infusion of miR-20a induces inflammation and motor neuron degeneration in uninjured spinal cord, whereas inhibition of miR-20a leads to improved neuronal survival and increasing neurogenesis (Jee et al. 2012). Other members of the MiR-17-92 cluster have been associated with inhibition of antiangiogenic genes such as thrombospodin-1 and connective tissue growth factor, suggesting a role in the control of neovascularization (Kuhnert and Kuo 2010). miR-92a, in particular, blocks angiogenesis in both in vitro and in vivo mice models of limb ischemia and myocardial infarction (Bonauer et al. 2009). The third miRNA, miR-30d, has been observed to control apoptosis (Li et al. 2016; Lv et al. 2018) and promote angiogenesis and tumor growth in an in vivo mouse model (Lin et al. 2017). All 3 have oncogenic involvement (Kobayashi et al. 2012; Mogilyansky and Rigoutsos 2013; Yang et al. 2013; Zhou et al. 2017; Huang et al. 2018; Kohram et al. 2018; Zhu et al. 2018; Che et al. 2019; Fahim et al. 2019) and associations with TBI (Sharma et al. 2014; Miao et al. 2015; Bhomia et al*.* 2016; Chandran et al. 2017). Additionally, miR-20a and miR-30d expression are both upregulated with exercise, in parallel to upregulation with TBI (Baggish et al. 2011; Makarova et al. 2014; Wang et al. 2017), whereas miR-92a is downregulated with exercise, in apparent opposition to its upregulation with TBI (Nielsen et al. 2014; Sapp 2015). While miRNA studies of brain injury models are still nascent, the aforementioned studies suggest that the elevated miRNA levels observed in the current cohort may be related to chronic neuroinflammatory processes. Future studies incorporating both age- and gender-matched nonathlete controls, as well as noncontact sports athlete controls will help to pinpoint whether the elevated preseason miRNA levels are due to neuroinflammation or peripheral musculoskeletal inflammation.

### rCBF in Putamen Mediates the Relationship Between miRNAs and VR Balance

We identified a 3-way interaction between rCBF, miRNA levels, and VR Balance in bilateral putamen. When the Sobel’s test revealed *P* < 0.05, the percent mediation effects ranged from 75% to 100% where rCBF in the putamen transmitted the effect of miRNA levels to VR Balance. All of the control mediations, which tested for the mediation effect of miRNA levels on the relationship between rCBF and VR Balance, had percent mediations of 15% or less. These findings highlight rCBF as a necessary variable for 2 separate miRNAs (miR-30d and miR-92a) to affect behavior and suggest a possible mechanism in place of overlapping associations (i.e., intermediary phenotype). In both hemispheres, clusters were localized to the rostral portion of the putamen but were relatively more dorsal in the left hemisphere and ventral in the right hemisphere. It is important to note that while the right-hemisphere cluster was primarily centered within the putamen, the left-hemisphere cluster was at the anterior and lateral border of the putamen and extended into white matter and cortical regions known to project more densely to the striosomes than to the matrix compartment of the putamen (Crittenden and Graybiel 2011). Whether this disparity is due to the predominant right-handedness of the present cohort (100% right-handed) or to some functional hemispheric difference within the putamen (Sainburg and Wang 2002) remains to be elucidated in a larger cohort.

The putamen, together with the caudate, form the striatum and are the input nuclei of the basal ganglia, which receive extensive direct afferents from the cortex. Recent works suggest that cortical inputs to the basal ganglia are organized both topographically and functionally, where the rostral putamen receives inputs relatively more densely from the prefrontal, orbitofrontal, and anterior cingulate cortices (Haber et al. 2006; Calzavara et al. 2007) and the caudal putamen receiving inputs mostly from parietal, temporal, presupplementary and supplementary motor areas (Yeterian and Pandya 1998; Lehericy et al. 2004). This organization may also relate to the rostral–ventral gradient of striosome versus matrix compartments in these nuclei (Crittenden and Graybiel 2011), which may be relevant to identifying the functional networks or systems impacted by HAEs. It is of note that the striosome-dominant cortical domains included in the left putaminal cluster associated with VR Balance and miRNAs (including INS, FOC, and subcallosal cingulate) have been shown to exhibit altered connectivity with the putamen in the genetic disorder, X-linked dystonia parkinsonism (Blood et al. 2018).

The striatal nuclei also receive input from the cerebellum via a disynaptic connection via the thalamus (Hoshi et al. 2005) and project back to the cerebellum also via a disynaptic connection via the subthalamic nucleus (Bostan et al. 2010). The cerebellum is known to be involved in postural control and balance (Coffman et al. 2011) and may work together with the basal ganglia to coordinate different subcomponents of such functions, in addition to other coordinating other nonmotor functions (reviewed in Bostan and Strick 2018). Although cerebellar perfusion related to levels of 2 miRNAs (miR-20a and miR-92a) evaluated in this study, this region was not directly correlated with motor or behavioral output measures. However, it is possible that abnormal perfusion in this region contributed in some less direct way to these measures.

The athletes in the current study had an average of 11 years of participation in contact sports. The fact that elevated miRNA levels were found at a timepoint when these athletes had at least 12 weeks of rest from impact practice suggests the presence of a chronic neuroinflammatory process unrelated to acute or recent head impacts. It should be noted that relationships of miRNA to VR Balance were not altered when years of football exposure were used as covariates, and the number of prior concussions was not predictive of miRNA levels or balance behavior, yet hypothesis generation analyses with the subsequent season of HAEs showed nominal effects (*P* < 0.05, uncorrected) between HAEs and 6 of 9 preseason miRNA’s and 3 Put rCBF measures. Further work is needed to explicitly connect these mediation results to quantitation of HAEs. While the miRNA levels were associated with widespread changes in rCBF within multiple brain regions, only rCBF in bilateral putamen was associated with the impaired VR Balance. These results provide preliminary evidence of the utility of an integrative systems neuroscience approach to study abnormal perturbations such as those caused by participation in impact sports and may have general applicability in other illnesses. In the future, abnormal miRNAs and VR Balance task performance might be used in return to play decision by identifying athletes that need additional recovery and/or treatment. Longer-term application includes the development of anti-inflammatory drugs and/or transcriptome or metabolome products that target the putamen to alleviate postural control impairment.

### Study Limitations and Assessment of Confounds

Although the results presented here were robust for miR-30d and miR-92a, some of the other results were not as strong, likely due to the modest cohort size, arguing for replication in a larger cohort. It is also important to note that the current cohort consisted of only male athletes, so we were unable to assess the possibility of gender differences. Future studies with females will be important to generalize these results across both genders.

An important research question that arises from the current study is whether there exists a dose–response to prior concussions. The current cohort only had 5 subjects with 1 and 2 subjects with 2 prior concussions and was therefore limited for such an investigation. Larger studies with a wider range of prior concussion diagnoses will improve our understanding of how prior concussion diagnosis relates to miRNA levels. Moreover, investigating the number and impact of HAEs via head accelerometers would also provide additional information about exposure to head trauma that should be considered in future studies. It should also be noted that this study used peripheral blood measures of miRNA as opposed to central nervous system-derived or enriched exosomal miRNA to test the hypothesis that centrally produced neuroinflammation is mediating the motor/cognitive and rCBF changes; further studies measuring centrally derived miRNA associated with inflammatory pathways would provide one avenue forward for verifying our findings.

To assess the possibility of confounds, we incorporated control mediation analyses that were predicted to be nonsignificant. In no case did miRNA levels act as a mediator to the relationship between rCBF and VR Balance scores, thereby serving as a consistent negative control. This assessment is crucial to understand the specific 3-way relationships we found in this study as others have argued that mediation analyses, which include 1) precedence information (i.e., miRNA is always the IV and behavior is the output variable) and 2) confounding controls to rule out nonspuriousness, allow better understanding of potential mechanisms of injury, even in a cross-sectional analyses that does not include longitudinal information or an intervention (Shrout and Bolger 2002; Cohen and Cohen 2003). Showing that regional brain imaging variables were needed for the effects of miRNA on behavior goes beyond the traditional construct of an intermediary phenotype, where the intermediary phenotype acts as a node in an overlapping set of associations. Per MacKinnon (2007), “mediating variables…transmit the effect of one variable to another variable,” which means that the mediator lies in the causal pathway between 2 other variables (i.e., miRNA—> perfusion— > behavior). The strongest mediation effects were between 75% and 100%, indicating regional Put measures filled a causally important role carrying the effect of miR-92a levels to balance behavior and miR-30d levels to balance behavior.

## Conclusion

In this study, we used a novel imaging-omics approach to study the relationship between rCBF, miRNA levels, and motor control behavior in NCAA Division I male football athletes. Mediation analysis revealed that rCBF is a significant mediator for the detrimental effects of abnormal inflammatory miRNA levels on motor control. This finding leads to the hypothesis that the presence of persistent elevations of inflammatory miRNAs in these athletes may be related to their history of participation in contact sports, despite 12 weeks without contact practice or games. This study offers preliminary evidence for the utility of an integrative systems neuroscience approach to study abnormal perturbations due to participation in contact athletics and suggests multiple applications to the study of other phenomena affecting the brain.

## Supplementary Material

ChenETAL_CerCor-2020-00020_SupplementalMaterials_tgaa078

## Notes

We thank the Pennsylvania State University football players for their effort participating in this study. We also thank Katie Finelli and Madeleine Scaramuzzo for their assistance with subject recruitment and data collection. Lastly, we thank the reviewers, whose guidance was substantial and beyond what most reviewers do; they deserve to be authors on this paper. *Conflict of Interest*: Drs L.P. and M.B. are inventors of a US patent application filed by Uniformed Services University of the Health Sciences (USUHS) regarding the potential utilities of selected miRNAs as diagnostic biomarkers for TBI. The other authors declare they have no financial or other conflicts of interest with regard to the data and analyses presented herein.

The opinions expressed herein are those of authors and are not necessarily representative of those of the USUHS, Department of Defense or, the United States Army, Navy, Air Force, and Defense Medical Research and Development Program (DMRDP), or of the institutions to which these authors belong.
