# Supplementary material for: Brain Perfusion Mediates the Relationship Between miRNA Levels and Postural Control
Source: Cereb Cortex Commun. 2020 Oct 26;1(1):tgaa078. doi: 10.1093/texcom/tgaa078 (PMC8153038; doi:10.1093/texcom/tgaa078)
Supplement: ChenETAL_CerCor-2020-00020_SupplementalMaterials_tgaa078 [file chenetal_cercor-2020-00020_supplementalmaterials_tgaa078.docx]

**Supplemental Methods:**An exploratory analysis relating HAEs collected across season to miRNA, rCBF, and VR behavior collected pre-season was performed. HAEs were monitored at all contact practice sessions (max = 53) using the BodiTrak sensor system from The Head Health Network (Slobounov et al., 2017). Sensors were mounted in each active player’s helmet prior to contact practice (no games were monitored). Sensor outputs included peak translational acceleration (PTA; G-units) and impact location. HAEs were quantified as 1) cumulative hits ≥25G & <80G (25G) and ≥80G (80G) (cHAE_25G_ and cHAE_80G_; Eq. 1) and 2) cumulative hits exceeding 25G and 80G normalized to the total number of sessions per player (aHAE_25G_ and aHAE_80G_; Eq.2). The G-unit thresholds (Th) were selected based on previous reports of impacts related to brain health and injury (MeCuen et al., 2015; Broglio et al., 2010).

$$\left( 1 \right) {cHAE}_{Th,i}= \sum_{k=1}^{N} u\left( {PTA}_{k,i}-Th \right)$$

$$where u\left( x \right)=\left\{ \begin{matrix} 1 if x>0 \\ 0 if x \leq0 \end{matrix} \right.$$

$$\left( 2 \right) {aHAE}_{Th,i}=\frac{{cHAE}_{Th,i}}{{sessions}_{i}}$$

**Suppl Methods References:**

S. M. Slobounov, A. Walter, H. C. Breiter, D. C. Zhu, X. Bai, T. Bream, P. Seidenberg, X. Mao, B. Johnson, T. M. Talavage, The effect of repetitive subconcussive collisions on brain integrity in collegiate football players over a single football seasonA multi-modal neuroimaging study. *NeuroImage Clin.* **14**, 708–718 (2017).

E. McCuen, D. Svaldi, K. Breedlove, N. Kraz, B. Cummiskey, E. L. Breedlove, J. Traver, K. F. Desmond, R. E. Hannemann, E. Zanath, A. Guerra, L. Leverenz, T. M. Talavage, E. A. Nauman, Collegiate women’s soccer players suffer greater cumulative head impacts than their high school counterparts. *J. Biomech.* **48**, 3720–3723 (2015).

S. P. Broglio, B. Schnebel, J. J. Sosnoff, S. Shin, X. Fend, X. He, J. Zimmerman, Biomechanical properties of concussions in high school football. *Med. Sci. Sports Exerc.* **42**, 2064–2071 (2010).

Supplemental Figure 1a) Clusters with significant associations between preseason CBF and preseason comprehensive VR score. Correlation plots extracted from these clusters are shown below. b) Clusters with significant associations between preseason CBF and preseason balance VR score. Correlation plots extracted from these clusters are shown below.


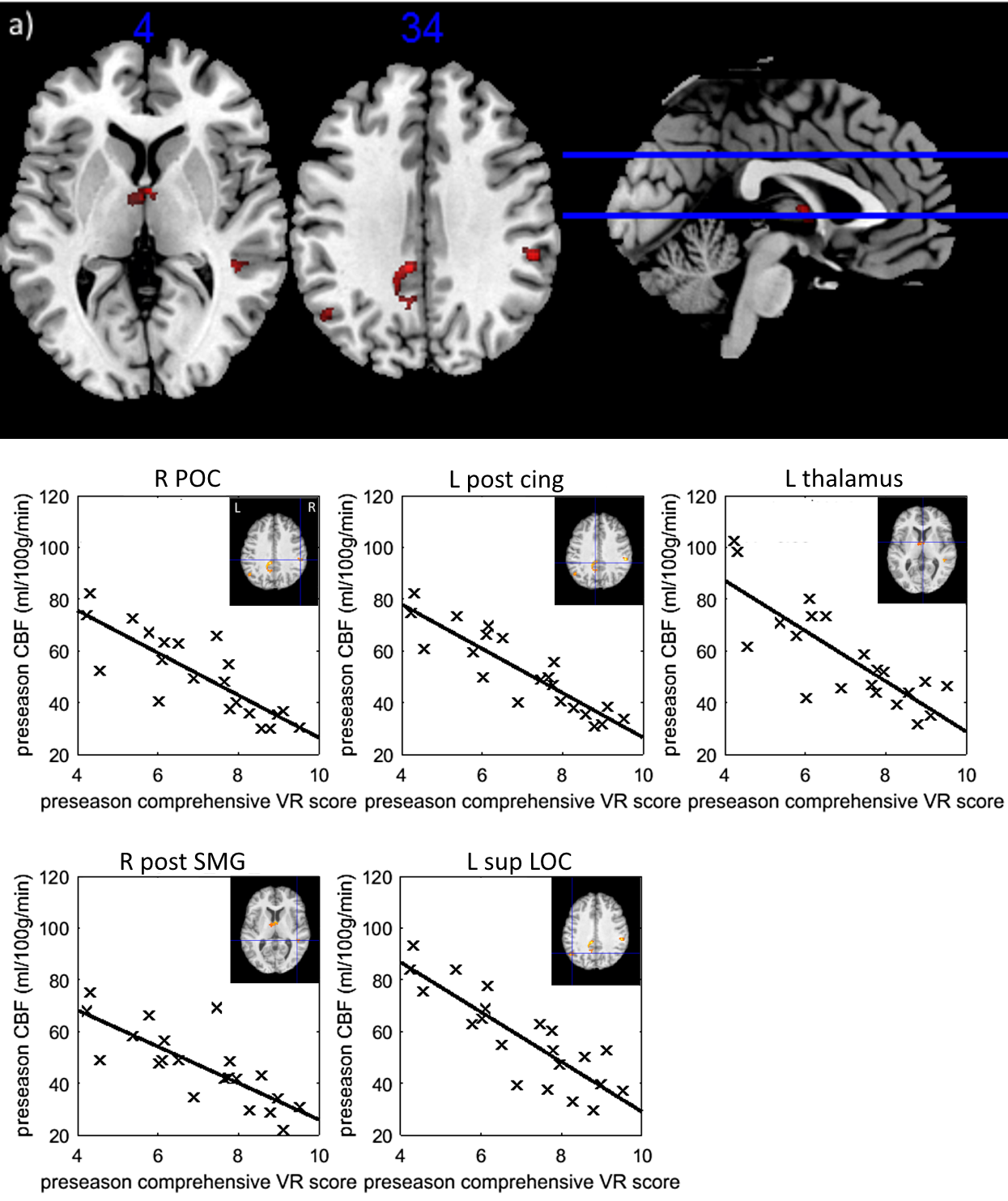


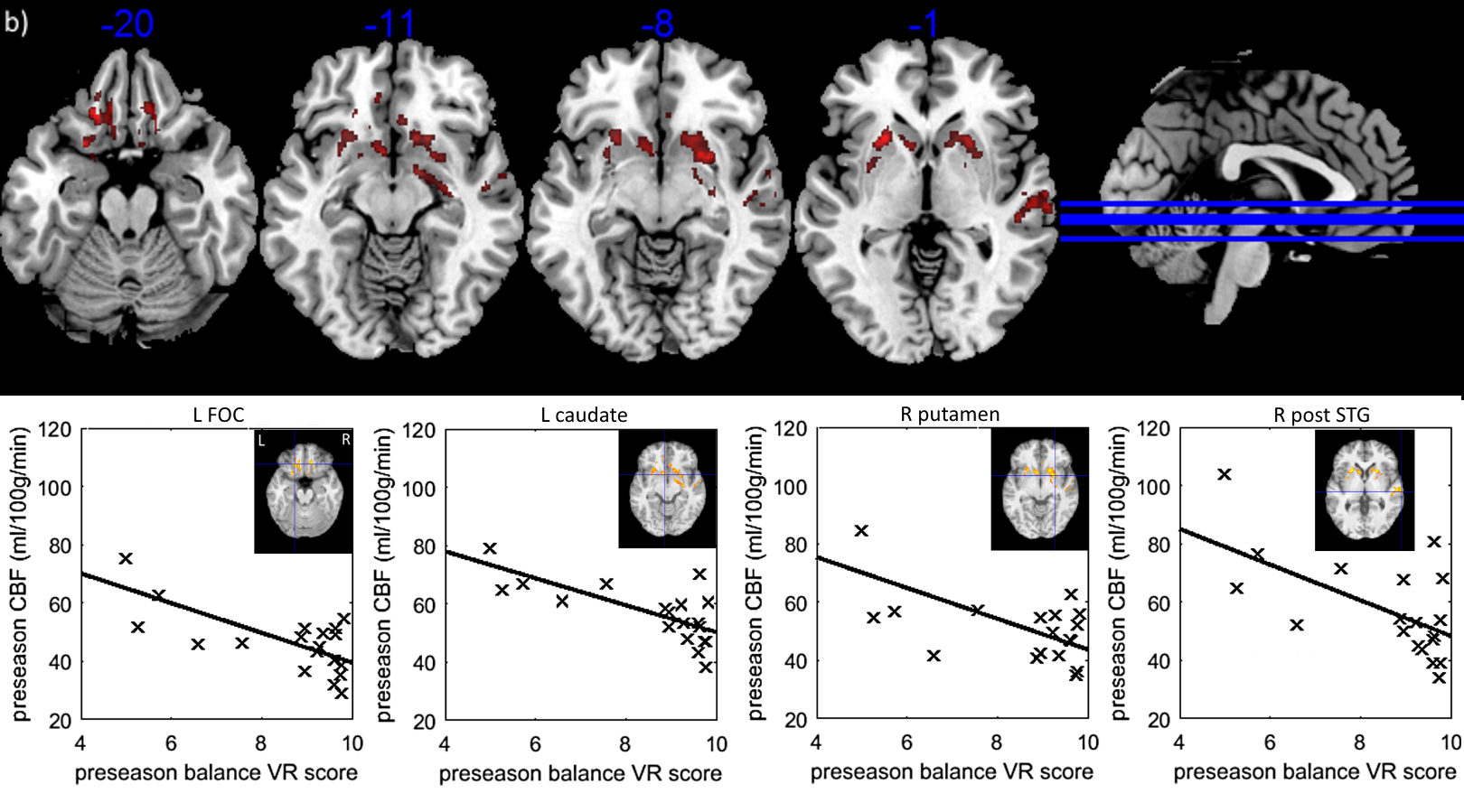


Supplementary Figure 2. Clusters with significant associations between preseason CBF and miRNA levels, overlaid onto a single subject’s high-resolution anatomical images.


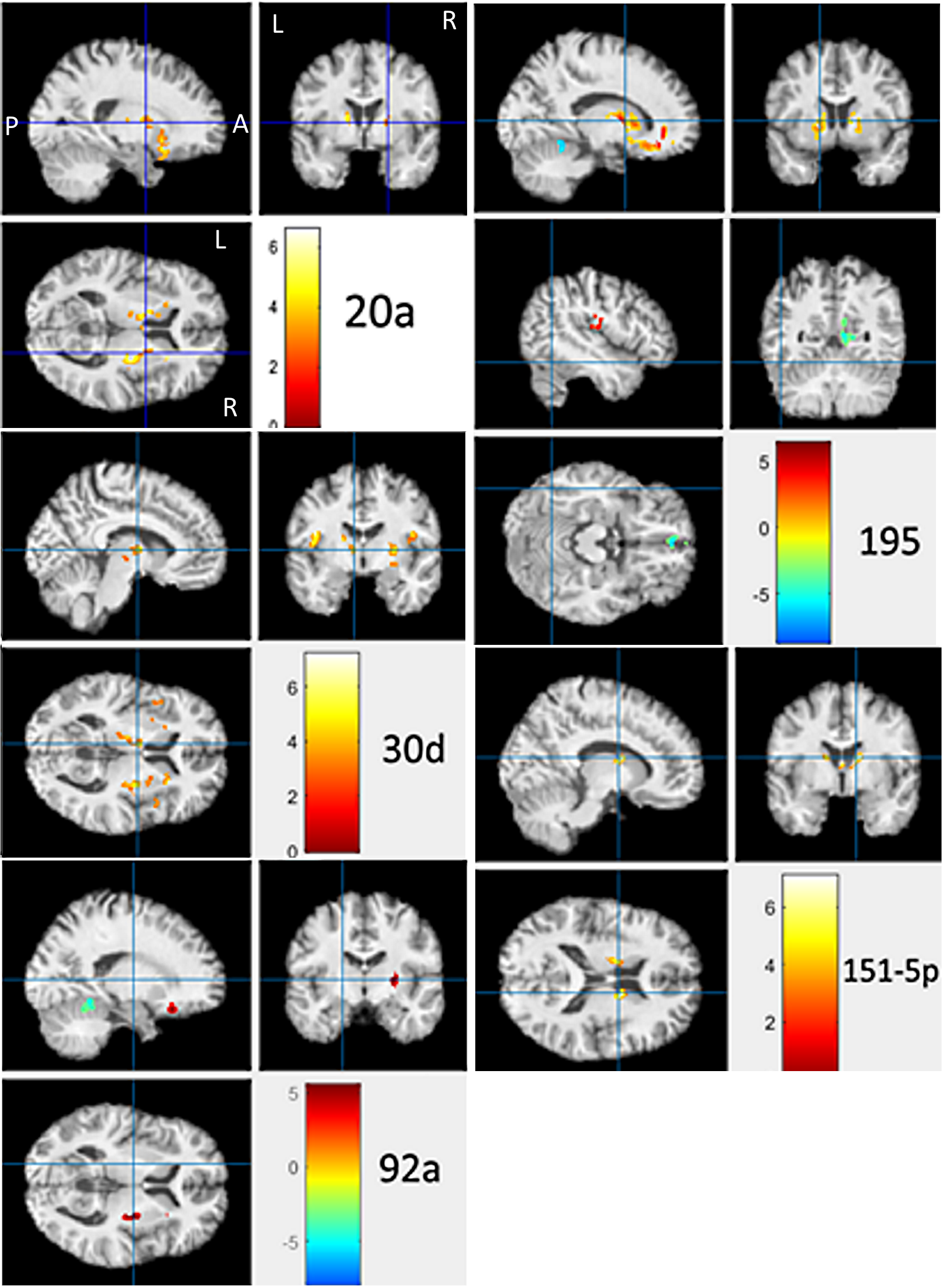


| miRNA | dir | Nvoxels | Peak T | p(unc) | x,y,z {mm} | region | label |
| --- | --- | --- | --- | --- | --- | --- | --- |
| miR-505* (k=200) | +ve | 1674 | 7.4 | 5.12E-07 | 26, -11, 6 | sub-lobar | R. Putamen (27%), R. Pallidum (10%), R. FOC (9%), R. Caudate (5%), R. Subcall (4%), R. POC (3%), R. Thalamus (2%), R. Insula (2%), R. Accumbens (2%) |
|  |  | 997 | 6.6 | 2.06E-06 | -18, 24, -21 | frontal | L. FOC(19%), L. Subcall (17%), L. Caudate (8%), L. Putamen (4%), L. Accumbens (4%), L. Paracing (2%), L. FMC (2%) |
|  | -ve | 278 | 7.6 | 3.91E-07 | -14, -51, -14 | ant. Cerebellum | L. V (57%), L. VI (35%), L. lingual (6%), L. I-IV (2%) |
| miR-486 (k=214) | +ve | 2437 | 11 | 1.21E-09 | 26, -17, 2 | sub-lobar | R. Putamen (21%), R. FOC(10%), R. Pallidum (8%), R. Subcall (5%), R. FMC (4%), R. Thalamus (3%), R. Frontal Pole (3%), R. Caudate (3%), R. POC (3%), R. Accumbens (2%), R. Insula (2%) |
|  |  | 2040 | 9.1 | 1.76E-08 | -11, 26, -11 | limbic | L. FOC(18%), L. Subcall(14%), L. Putamen (10%), L. Caudate (8%), L. Pallidum (3%), L. Thalamus (3%), L. Paracing (2%), L. Accumbens (2%) |
|  |  | 445 | 7.9 | 1.57E-07 | 0, -30, -10 | midbrain | Brain-Stem (87%) |
|  | -ve | 314 | 6.5 | 2.13E-06 | -21, -57, -20 | limbic | L. V (52%), L. VI (46%), L. Lingual (2%), L. temp. occ. Fusiform (1%) |
|  |  | 357 | 5.2 | 3.18E-05 | -32, 29, -1 | frontal | L. FOC (82%), L. Frontal Pole (4%), L. Temporal Pole (4%), L. Insula (2%) |

Supplemental Table 1. Clusters with significant correlations between preseason CBF and miRNA levels of miR-505* and miR-486. These results did not survive Cook’s Distance analysis and were excluded from three-way and mediation analyses. They are included here for reference only.
